# Mitochondrial Dysfunction in Chemotherapy-Induced Peripheral Neuropathy (CIPN)

**DOI:** 10.3390/toxics3020198

**Published:** 2015-06-05

**Authors:** Annalisa Canta, Eleonora Pozzi, Valentina Alda Carozzi

**Affiliations:** Experimental Neurology Unit, Department of Surgery and Translational Medicine, University of Milan-Bicocca, Via Cadore 48, 20900 Monza (MB), Italy; E-Mails: e.pozzi18@campus.unimib.it (E.P.); valentina.carozzi1@unimib.it (V.A.C.)

**Keywords:** Chemotherapy compounds, peripheral neurotoxicity, neuropathic pain, mitochondria, mitotoxicity

## Abstract

The mitochondrial dysfunction has a critical role in several disorders including chemotherapy-induced peripheral neuropathies (CIPN). This is due to a related dysregulation of pathways involving calcium signalling, reactive oxygen species and apoptosis. Vincristine is able to affect calcium movement through the Dorsal Root Ganglia (DRG) neuronal mitochondrial membrane, altering its homeostasis and leading to abnormal neuronal excitability. Paclitaxel induces the opening of the mitochondrial permeability transition pore in axons followed by mitochondrial membrane potential loss, increased reactive oxygen species generation, ATP level reduction, calcium release and mitochondrial swelling. Cisplatin and oxaliplatin form adducts with mitochondrial DNA producing inhibition of replication, disruption of transcription and morphological abnormalities within mitochondria in DRG neurons, leading to a gradual energy failure. Bortezomib is able to modify mitochondrial calcium homeostasis and mitochondrial respiratory chain. Moreover, the expression of a certain number of genes, including those controlling mitochondrial functions, was altered in patients with bortezomib-induced peripheral neuropathy.

## 1. Introduction

Cancer is a serious disease that afflicts the world population; fortunately, there are different useful drugs used as first-line treatment of solid common tumours (*i.e.*, lung, colorectal, breast and gastric cancers) and blood cancers. Among these chemotherapeutic drugs, DNA-alkylating agents (platinum derivatives like cisplatin and oxaliplatin), anti-tubulin compounds (Taxanes like paclitaxel and Vinca alkaloids like vincristine) and proteasome inhibitors (bortezomib) are generally successfully employed. All these compounds are different in nature, chemical structures and also in their mechanism and site of action, but they have a common and relevant side effect that is the onset of a peripheral neurotoxicity frequently associated with the development of neuropathic pain (Table 1). Patients affected by chemotherapy-induced peripheral neuropathy (CIPN) often manifest symptoms that may be disabling, influencing their daily activities and compromising their life quality. Finally, these side effects can lead to the reduction, discontinuation or even interruption of the chemotherapeutic treatment. Generally, neurological complication of cancer chemotherapy involves a chronic and distal sensory axonal peripheral neuropathy; patients suffering from this condition report numbness, dysesthesia, paraesthesia, sensory loss and tingling in the affected regions [1,2]. Chemotherapy-induced neurotoxicity preferentially takes place in Dorsal Root Ganglia (DRG), sensory neurons, satellite cells and Schwann cells. Even if different studies support the hypothesis that CIPN pathogenesis is related to the development of axonopathy (through dying back axonal damage) and neuronopathy (with the involvement of neurons’ cell body of DRG), the precise pathophysiology is not clearly understood. Various and different underlying mechanisms involved in the onset of peripheral neuropathy have been investigated and suggested for the frequently used classes of chemotherapeutic compounds. The most common mechanisms proposed are nuclear DNA (nDNA) damage [3], altered axonal transport [4,5,6], microtubule changes [7,8], endoplasmic reticulum integrity alteration [9], dysfunctions in sodium (Na^+^), calcium (Ca^2+^) and potassium (K^+^) channels [10,11,12,13], Ca^2+^ signalling changes [14], modifications of peripheral vascularization [15,16], changes in the expression of Transient Receptors Potentials as well as in molecules implicated in the glutamate signalling [17,18,19,20], reactive oxygen species (ROS) production [21,22,23,24] and mitochondrial function impairment [25,26,27]. Even if the investigation of all these mechanisms is interesting for improved understanding of the pathophysiology of CIPN, this review aims to highlight how a mitochondrial damage and the impairment of mitochondrial function may be responsible for neuropathy onset and its development.

## 2. Mitochondria and Mitotoxicity

Mitochondria are organelles, localized in eukaryotic cells cytoplasm, with their own circular mitochondrial DNA (mDNA) that encodes for 13 proteins. They are involved in mitochondrial electron transport chain (mETC) subunits synthesis and implicated in cellular energy production [28]. Mitochondria are complex machines and their correct function is essential for the preservation of several inter-related pathways such as apoptotic signalling pathways [29], intracellular Ca^2+^ regulation [30] and ROS generation [31]. They are also involved in cell death; for example, it was demonstrated that proteins such as Cytochrome c (Cyt c) are implicated in the apoptotic processes [32]. In response to different apoptotic stimuli, Cyt c is released into the cytosol with a consequent caspase 9 activation and interaction with apoptotic protease activating factor-1 (Apaf-1) and ATP [33]. Moreover, other proteins involved in the apoptotic process are released from mitochondria like procaspases and Smac-α/DIABLO [32].

**Table 1:** Paclitaxel, cisplatin, oxaliplatin, vincristine and bortezomib: chemical structures, principal mechanism of action and peripheral neuropathy.

Drugs	Principal mechanism of action	Peripheral neuropathy
**PACLITAXEL**	Impairment of microtubules dynamic	SENSORY
**CISPLATIN**	Interaction with DNA	SENSORY PROPRIOCEPTIVE
**OXALIPLATIN**	Interaction with DNA	SENSORY PROPRIOCEPTIVE
**VINCRISTINE**	Impairment of microtubules dynamic	SENSORY MOTOR PAINFUL
**BORTEZOMIB**	Inhibition of proteasome activity	SENSORY PAINFUL

Over the past decade, an important role in mitochondria degeneration was attributed to mitochondrial multi-molecular complex called mitochondrial Permeability Transition Pore (mPTP), a high conductance channel in the inner membrane, permeable to solutes up to 1.5 kDa. mPTP contains different trans-membrane proteins like adenine nucleotide translocator (ANT), mitochondrial phosphate carrier (PiC), voltage-dependent anion channel (VDAC), and cyclophilin-D (Cyp-D) that are directly or indirectly involved in the formation/opening of the pore [34,35].

mPTP is a Ca^2+^, pH, redox, adenine nucleotide and voltage sensitive channel so that, under particular conditions, e.g., in the presence of adenine nucleotide depletion, mitochondrial Ca^2+^ overfilling, elevated levels of free PO_4_^3−^, or oxidative stress, it opens. The mPTP opening causes different events: collapse of mitochondrial membrane potential, reduction in ATP level, increase in ROS, Ca^2+^ release, vacuolization and mitochondrial swelling with final cell death [36].

Furthermore, in 2001, Zamzani and Kroemer proposed an alternative hypothesis for mitochondrial permeabilization according to which the pro-apoptotic protein Bcl-2 is involved in the formation of the mitochondrial membrane pores by oligomerization [33].

In the peripheral nervous system, it was demonstrated that more than 90% of mitochondria are localized in the axons; since axonal mitochondria are fundamental for energy generation in axons, a defect in mitochondrial energy metabolism can cause axonal transport degeneration and nerve failure [1].

In the last few years, several *in vivo* and *in vitro* models of CIPN focused the attention on the “mitotoxicity hypothesis” which supports the impairment of mitochondrial functions due to damage to primary afferent sensory neurons [25,37,38,39]. Consistent with this hypothesis, morphological and functional alterations of mitochondria appear to be involved in CIPN. Moreover, the results of mitochondrial toxicity are more evident in regions with high metabolic request, like primary afferent sensory neurons, where an elevated mitochondria concentration is present [40]. In neurons, mitochondrial damage may induce bioenergetic deficits that modify the correct functionality of voltage-gated Na^+^ channel leading to an altered neuron ability to synthetize ion transporters. All these alterations contribute to membrane depolarization and abnormal spontaneous discharges followed by degeneration in primary afferent sensory neurons and intraepidermal nerve fibers (IENFs) [41,42].

*In vivo* studies demonstrated that rats treated with paclitaxel 2 mg/kg and Vincristine 50 µg/kg for four or 10 alternate days, respectively, developed peripheral neuropathy with abnormal spontaneous discharge in about 20%–35% and 15% of sensory C and A fibers, respectively [41,43]. Moreover, microscopic analysis performed on peripheral nerve sensory axons of rats treated with paclitaxel evidenced a lot of vacuolated and swollen mitochondria, phenomena also present in lumbar DRG of paclitaxel-treated rats [25,38,44]. These damages precede neuronal apoptosis that may involve different pathways such as the activation of caspase cascade, the dysregulation of intracellular Ca^2+^, the damage of mDNA, the alteration of mETC and membrane potential, the loss of enzymes involved in antioxidant activities [25,26,44]. Furthermore, it has been demonstrated that an ineffective mitophagy leads to dysfunctional mitochondria accumulation, causing an oxidative damage with ROS and reactive nitrogen species (RNS) increase, which in turn creates mitochondrial dysfunctions [44]. Mitochondrial permeability alterations were observed in several neurodegenerative disorders in relation to oxidative stress, elevated free PO_4_ level, mitochondrial Ca^2+^ overfilling accompanied by adenine nucleotide depletion [45].

Moreover, studies conducted by Züchner and colleagues in 2004 demonstrated that mutations in the mitochondrial GTPase mitofusin 2, a gene involved in the mitochondrial homeostasis, are implicated in the onset of hereditary axonal peripheral neuropathies as Charcot-Marie-Tooth type 2 [46].

This paper reviews the role of mitochondria damage in the onset, development and severity of CIPN.

## 3. Paclitaxel

Paclitaxel (Taxol^®^) is an antineoplastic drug derived from “*Taxus brevifolia*” tree bark [47] and commonly used for the treatment of several solid tumors such as breast, ovarian and lung cancers. Paclitaxel is a microtubule-binding compound that is able to cross plasmatic membrane through passive diffusion and bind the *N*-terminal region of β-tubulin monomer of microtubules. The specific and reversible paclitaxel interaction with microtubules leads to enhanced microtubule polymerization and decreased microtubule depolymerization, moving microtubules’ equilibrium towards the polymeric structure [48,49,50,51]. The result is the arrest of cellular mitosis at the metaphase-anaphase transition (G2/M phase) inducing cancer cells death by apoptosis [50,52]. Paclitaxel is active against proliferating cells like cancer cells but also neurons (that are not able to proliferate) are susceptible to paclitaxel treatment.

### 3.1. Paclitaxel-Induced Peripheral Neuropathy

Paclitaxel may induce the onset of neuropathy characterized by paraesthesia and sensory loss, frequently associated with the development of neuropathic pain [1]. Patients affected by paclitaxel-induced peripheral neurotoxicity often manifest symptoms that may be disabling and compromise their quality of life. Paclitaxel-induced peripheral neurotoxicity produces different symptoms like tingling and numbness, on-going burning pain, cold and mechanical allodynia. The sensory system is always affected, while the motor system is generally less compromised [43,53]. Neuropathic symptoms usually start in the feet in symmetrical way, but also the hands can be affected in the dying-back neuropathy, in which the distal sensory axons degenerate at an early stage [53,54].

Both *in vitro* and *in vivo* studies evidenced that paclitaxel chronic treatment induces axonal degeneration followed by secondary demyelination and nerve fibre loss in the presence of a severe peripheral neuropathy [55]. Moreover, a dose dependent reduction in neurite length and changes in morphology of DRG sensory neurons were also observed [56]. The authors hypothesized that the reduction in neurite length is due to a direct interaction between paclitaxel and the axon with the development of axonal degeneration through local mechanisms [57]. These results suggested that the severity and incidence of CIPN depend on the cumulative doses of paclitaxel [58].

### 3.2. Paclitaxel and Mitochondria

Even if in the last few years mitochondria were identified as active players in paclitaxel-induced axonal degeneration, the mechanism remains to be elucidated.

Numerous *in vitro* experiments have evidenced that paclitaxel is able to alter mitochondrial structure and function; in fact, studies performed in non-neuronal tumor cells and cultured brain stem neurons showed that paclitaxel treatment evokes immediate mitochondrial depolarization and Ca^2+^ release from mitochondria, due to the opening of mPTP [59,60,61]. It has been demonstrated that mitochondria are implicated in intracellular Ca^2+^ homeostasis: an increased Ca^2+^ efflux is observed after paclitaxel treatment [60]. Furthermore, since intramitochondrial Ca^2+^ signal regulates different mitochondrial functions like Ca^2+^-dependent dehydrogenases, a reduction in ATP production and, as a consequence, an alteration of other cell functions can occur [62].

In 2012, Jaggi and collaborators confirmed that paclitaxel-induced changes in mitochondrial structure were related to an increase in Ca^2+^-mediated neuronal excitability [2].

Moreover, in 2000, Andrè and colleagues performed *ex vivo* studies using mitochondria isolated from human neuroblastoma cells treated with paclitaxel, demonstrating that paclitaxel is able to act by inducing the release of Cyt c that activates caspase signalling pathway during apoptosis [63]. The release of Cyt c is blocked by cyclosporin A, a mPTP inhibitor, which prevents mPTP opening, providing further evidence that paclitaxel directly acts on mitochondria membrane, independently of its direct interaction with microtubules [64].

In 2002, Carrè and colleagues suggested that paclitaxel is able to bind to mitochondria through a direct interaction with β-tubulin, that is specifically bound to mPTP offering a binding site for paclitaxel-mitochondria interaction; the results of mPTP opening are vacuolization and mitochondria swelling with functionally compromised mitochondria [25,63,64]. Since paclitaxel-induced vacuolated and swollen mitochondria were observed in rat kidney, heart and brain [65] as well as in human neuroblastoma cells [63], it may be hypothesized that these phenomena are not tissue-specific events, suggesting that paclitaxel may induce mitochondria degeneration also in peripheral sensory axons [25].

In addition, ELISA assays performed by Rodi and co-workers in 1999 confirmed that the mitochondrial anti-apoptotic protein Bcl-2 is a paclitaxel-binding protein; *in vivo* studies showed that paclitaxel treatment leads to Bcl-2 inactivation with consequent protein phosphorylation and activation of the apoptotic cascade, suggesting that the apoptotic pathway may involve the binding of paclitaxel to Bcl-2. These results proposed that a reasonable mechanism for paclitaxel-mPTP interaction may exist and that it might be either tubulin dependent or independent [66].

In 2006, Flatters and Bennett conducted *in vivo* studies in which they evaluated the development of an evoked painful peripheral neuropathy due to paclitaxel treatment. They observed numerous swollen and vacuolated mitochondria in the axons of peripheral nerves of treated rats, confirming functional damage of peripheral nerve mitochondria [25]. Furthermore, the increasing number of degenerated mitochondria induced by paclitaxel treatment was found only in the sensory axons of dorsal root ganglia, supporting the presence of sensory but not motor dysfunctions [43].

After paclitaxel treatment, the electron gradient across the mitochondrial inner membrane is altered because of the swollen and vacuolated mitochondria. The presence of damaged mitochondria supports the mitotoxicity hypothesis, which suggests that paclitaxel produces a chronic sensory axonal energy deficiency that may be the primary cause of peripheral neuropathy features and reduction of the intraepidermal nerve fibres density [25,67].

**Figure 1:**
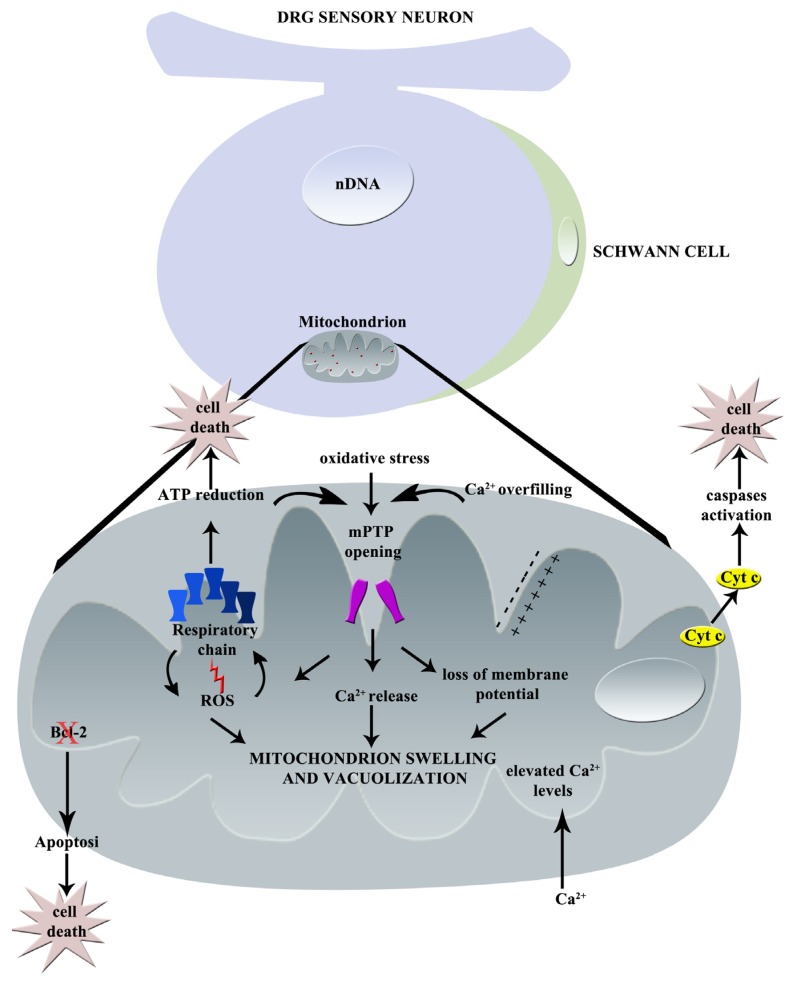
Graphical summary of paclitaxel-induced mechanisms of neurotoxicity: principal effects on mitochondrion (nDNA = nuclear DNA, mPTP = mitochondrial Permeability Transition Pore, Cyt c = Cytochrome C, Ca^2+^ = calcium, ROS = Reactive Oxygen Species).

In 2011, Zheng and co-workers performed experiments in *ex vivo* preparations of sciatic nerve axons collected from paclitaxel-treated rats in which they measured mitochondrial respiration and ATP production. They observed important deficits in maximally stimulated Complex I and Complex II-mediated respiration respectively, associated with significant deficit in ATP production [38]. Moreover, they observed that a prophylactic treatment with Acetyl-L-Carnitine (ALC) is able not only to inhibit the onset of paclitaxel-induced peripheral neuropathy and neuropathic pain but also to prevent mitochondria energy failure. These results showed that the effect of ALC may be due to an anti-mitotoxicity action suggesting mitotoxicity hypothesis as a plausible cause of sensory peripheral neuropathy induced by chronic treatment with paclitaxel [38].

In Figure 1, the principal effects of paclitaxel on mitochondria are represented.

## 4. Platinum Compounds

Platinum compounds belong to a family of platinum (Pt)-based anticancer drugs that includes several generations of compounds that have been developed over time in order to reduce toxicity and at the same time to enhance their anticancer activity: the first studies on cisplatin led to the second-generation carboplatin and subsequently third-generation oxaliplatin). Therefore, these compounds differ in chemical structure and are used in clinical practice to treat several types of solid tumors (e.g., lung, colorectal, breast, ovarian and testicular cancers) [68,69].

Although their toxicity profile differs, these drugs have a similar mechanism of action and cause some common symptoms. Patients may suffer from neuropathic pain and sensory loss, which represents their dose-limiting toxicity.

Regarding the clinical incidence of neuropathy, it has been observed that in patients, carboplatin evokes CIPN not only with much less frequency but also with less severity compared to cisplatin and oxaliplatin. However, at higher doses, it shares the same neurotoxic symptoms with other Pt anticancer drugs [69,70].

All Pt compounds are alkylating agents that bind the DNA double strand by crosslinks creating Pt-DNA adducts. This can interfere with DNA synthesis by inhibiting or slowing the duplication process, leading to cell cycle arrest, apoptosis and cell death.

Peripheral neuropathy is one of the main and most severe side effects evoked by these compounds due to Pt accumulation in the DRG because of the lack of a blood-brain barrier [44,69,71].

Pre-clinical studies, such as Pt concentration assay in DRG, morphological and morphometric analysis on DRG neurons and sensory nerve conduction velocity (NCV) measure, have shown an impairment of the peripheral nervous system following Pt antineoplastic drug treatment [20,72]. In fact, an increased amount of Pt-DNA adducts, a decrease in the size of soma, nucleus and nucleolus and a reduced NCV were observed in DRG neurons of cisplatin or oxaliplatin treated animals. Particularly, an involvement (impairment) of voltage-dependent channels represents an onset mechanism of oxaliplatin-induced neuropathy [73,74,75].

The severity of neurotoxicity is dose and time dependent. Additionally, genetic polymorphisms could increase or decrease the susceptibility to this side effect [76,77,78].

### 4.1. Cisplatin

Cisplatin was the first Pt compound to be synthesized and used, primarily in the treatment of metastatic ovarian and testicular cancers. Cisplatin is also known as *cis-dichloro-diammine-platinum*, as it is composed of a central Pt atom coordinated with two ammonia groups (NH_3_) and two chloride atoms (Cl) in *cis* position.

Like other Pt agents, its mechanism of action is based on the inhibition of DNA synthesis by formation of Pt adducts inter and intra-strands; in particular, the most prevalent adducts generated by cisplatin are guanine-guanine intra-strands [79]. High doses of cisplatin cause nephrotoxicity, a side effect that has been overcome with intravenous pre-hydration and the use of diuretics in patients [73]. Moreover, ototoxicity (sometimes irreversible) and bland hematologic toxicity are also observed in patients under treatment with cisplatin [69].

#### 4.1.1. Cisplatin-Induced Peripheral Neuropathy

About 30%–50% of patients treated with cisplatin are affected by peripheral neurotoxicity which becomes significant at cumulative doses around 300–400 mg/m^2^ [75,80,81]. This side effect usually appears in most patients between three and six months after drug administration and endures even after interruption [82]. Neuropathy is prevalently characterized by distal painful paresthesia and, at high doses (cumulative dose 600 mg/m^2^), by severe sensory ataxia [69,75]. Because of this side effect, approximately 20% of patients treated with cisplatin fails to complete anticancer therapy [83].

#### 4.1.2. Cisplatin and Mitochondria

Several studies have shown different cisplatin effects on mitochondria, such as mDNA-Pt adducts formation, incorrect mitochondrial protein synthesis, ROS generation and apoptosis via mitochondrial pathway induction.

Through PCR assay, Podratz and colleagues in 2011 demonstrated that cisplatin binds mDNA in DRG neurons at the same rate as in nDNA. However, unlike nDNA, mDNA does not have any DNA repair system (Base Excision Repair, BER, and Nucleotide Excision Repair pathway, NER); thus Pt adducts cannot be removed, interfering with replication and transcription of mDNA. This causes problems in mitochondrial protein synthesis, impairing the mitochondrial respiratory chain functionality [80]. At last, energy failure within DRG mitochondria results, in neurotoxicity.

The involvement of cisplatin in mitochondrial respiratory impairment was evidenced in an *in vitro* study conducted by Garrido and colleagues in 2008 in which they examined indices of mitochondrial activity noticing a decrease in respiratory control ratio (*i.e.*, the ratio between respiration State III and State IV, RCR) cisplatin-exposed mitochondria isolated from liver cells. The same study also confirmed the decrease in mDNA expression both *in vitro* as *in vivo* experiments in a dose- and time-dependent manner (these results occur for high doses of cisplatin) [84].

These evidences were subsequently verified by an *in vitro* study performed by Podratz and collaborators in 2011 on DRG culture exposed to cisplatin. Through BrdU (intercalator of replicating DNA) assay they observed that, due to Pt adducts, cisplatin is able to inhibit mDNA replication and transcription in dissociated DRG neurons. In addition, microscopic analysis showed that mitochondria within DRG neurons appear damaged, smaller and vacuolated after cisplatin exposure. The same morphological observations were evident in DRG and renal mitochondria of cisplatin-treated mice [80,85].

It is widely known that mitochondrial dysfunctions induce cellular oxidative stress; for this reason, cisplatin exposure can generate ROS resulting in oxidative damage in cells, including DRG neurons that are the main site of Pt accumulation [71,86,87,88].

Thus, in 2008, Jiang and collaborators investigated the mechanism of mitochondrial ROS generation in DRG produced by cisplatin, through the measurement of ROS accumulation in dissociated DRG sensory neurons incubated with different cisplatin concentrations. The data showed an increase in ROS production in a dose-dependent manner [87].

A similar study conducted by Marullo and co-workers in 2013 showed an increase in mitochondrial ROS levels correlated to mDNA damage in lung and prostate cancer cells during cisplatin exposure, demonstrating that this Pt compound is able to generate cytotoxicity also in other cell types and not only in neurons [88].

Moreover, cisplatin acts on apoptotic mitochondrial pathway through Cyt c-release and caspases activation as it was previously demonstrated by Gill and colleagues in 1998. In fact, using an immunoblotting analysis, they showed a significant increase in caspase 3 levels, an apoptotic marker, in cultures of DRG incubated with cisplatin compared with untreated cultures [89].

Furthermore, several successive studies explained this observation as a possible mechanism of pathogenesis of cisplatin-induced neuropathy [86,90].

Investigating the apoptotic process, McDonald and Windebank in 2002 discovered that cisplatin induces apoptosis not via extrinsic pathway mediated by the protein Fas but via mitochondrial intrinsic pathway. In fact, by immunofluorescence assay, they monitored the Cyt c-release, demonstrating that its translocation significantly increases out of mitochondria of cultured neuronal cells after drug exposure [91].

In 2007, Cullen and colleagues supposed that Cyt c-release could be enhanced by mPTP alteration: in particular, by interaction between Pt adducts and VDAC (a structural element of mPTP) [92].

Also, other reports demonstrated an involvement of apoptosis and mitochondrial pathways in other cisplatin related toxicities.

Among these studies, Park and collaborators in 2002 revealed that cisplatin induces activation of caspase 9 in a dose-dependent manner in cultures of renal cells leading to apoptosis and caspase 3 cleavage. Consequently, considering the Cyt c-localization, they observed that cisplatin also promotes Cyt c-release correlated with drug exposure time [90].

Based on these observations, Melli and collaborators in 2008 tested alpha-lipoic acid (aLA), a biological antioxidant, with the aim of preventing cisplatin-evoked neuropathy in preclinical models. The antioxidant and anti-apoptotic activity of this compound proved to be neuroprotective acting on mitochondria: the rate of dissociated DRG with damaged mitochondria exposed to cisplatin+aLA significantly decreases compared to DRG treated only with cisplatin [26]. Melli and collaborators hypothesized that cisplatin-induced oxidative stress was mediated by a downregulation of frataxin, a protein responsible for detoxification processes. In fact, frataxin deficiency is related to alterations of mitochondrial DNA, reduced oxidative phosphorylation and impaired antioxidant endogenous mechanisms [93].

**Figure 2:**
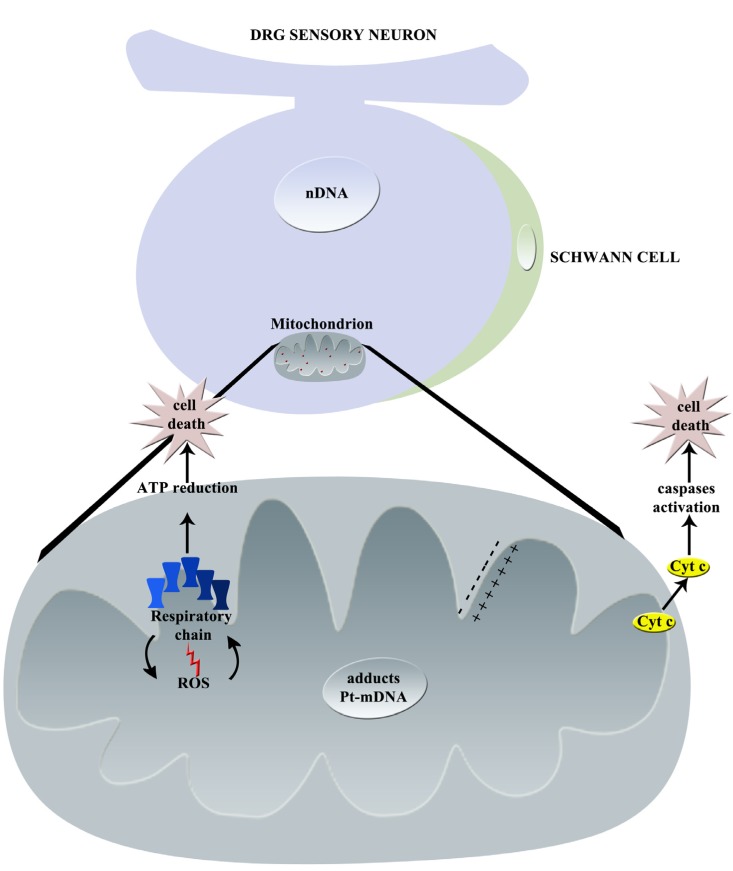
Graphical summary of cisplatin-induced mechanisms of neurotoxicity: principal effects on mitochondrion (nDNA = nuclear DNA, Pt = platinum, mDNA = mitochondrial DNA, Cyt c = Cytochrome C, ROS = Reactive Oxygen Species).

The principal effects of cisplatin on mitochondria are summarized in Figure 2.

### 4.2. Oxaliplatin

Oxaliplatin is a third generation Pt drug used as drug of choice in first line therapy alone or in combination with 5-fluorouracil and folic acid or as adjuvant, for the treatment of metastatic colorectal cancer. It is also employed for ovarian and pancreatic cancer in case of cisplatin-resistance [68,74]. Oxaliplatin is composed of a central Pt atom that forms a complex with 1,2-*diaminocyclohexane (DACH)* and an oxalate group.

Although to a lesser extent than cisplatin (three time less), oxaliplatin is able to produce Pt-adducts in DNA strands (both drugs mainly create guanine-guanine intra strands). For this reason, oxaliplatin generates less neurotoxic than cisplatin; in fact, it has been observed that the amount of Pt-adduct is linear with the severity of peripheral neuropathy (few adducts generate less toxicity) [14,69,71,94]. Hematologic toxicities (neutropenia and thrombocytopenia) are also detected during oxaliplatin treatment.

#### 4.2.1. Oxaliplatin-Induced Peripheral Neuropathy

As with other Pt compounds, neurotoxicity is the dose-limiting toxicity of oxaliplatin, of which it is possible to identify two different forms, an acute and a chronic form, respectively. The acute form arises during drug infusion and it is usually transient (reversible within a week), characterized by paresthesia and dysesthesias especially in the extremities; this form affects approximately 90% of patients treated with oxaliplatin [14,77,82,95]. Differently, the chronic form is a chronic peripheral neuropathy, a distal sensory neuropathy that arises after cumulative doses of 400–800 mg/m^2^, resolves in several months (even up to eight months or permanent), and is most similar to that which occurs for others Pt compounds [82,96]. Approximately, 90% of patients treated with oxaliplatin develop the peripheral neurotoxicity: 40%–50% of patients with Grade >2 while in 10%–20% of patients results >3. Therefore, neuropathy alters patients’ quality of life, leading to treatment discontinuation [75,77,96].

#### 4.2.2. Oxaliplatin and Mitochondria

The oxaliplatin effects on mitochondria have not been deeply investigated yet and remain partly unclear.

As it belongs to Pt compounds’ family, oxaliplatin creates mDNA adducts which, interfering with mitochondrial protein synthesis, can generate several mitochondrial abnormalities [44].

A rat model of oxaliplatin-evoked peripheral neuropathy showed an increase in mitochondrial morphological changes in saphenous nerves of treated animals compared to controls: in fact, mitochondria appeared swollen and vacuolated as happened in mitochondrial damage [37].

Besides, Zheng and co-workers in 2011 tested the mitochondrial functions *in vivo.* Mitochondrial respiratory and ATP assays were performed on isolated sciatic nerves, demonstrating a deficit in respiration rate in both Complex I and II of mETC followed by a decrease of ATP production in oxaliplatin-treated rats [38].

In order to confirm an mETC injury induced by oxaliplatin, Xiao and colleagues in 2012 conducted a study on rats through which they demonstrated, using behavioral tests, that the administration of complex-respiratory chain’s inhibitors such as rotenone, oligomycin and auranofin was able to aggravate the nervous system damage with additional mitochondrial activity impairment [97].

Moreover, a recent study by Kelley and co-workers in 2014 demonstrated that the exposure to high oxaliplatin concentrations increases the mitochondrial ROS levels in neuronal cultures, proving that a mitochondrial oxidative stress may play an important role in the pathogenesis of oxaliplatin-induced peripheral neuropathy [98].

Furthermore, as it has been observed in cisplatin reports, several studies noticed that also oxaliplatin is able to enhance the mitochondrial apoptosis.

Arango and colleagues in 2004 studied Bax (a member of pro-apoptotic Bcl-2 family that has a role in Cyt c-release) and Cyt c localization in cultured colon carcinoma cells under oxaliplatin exposure. They observed an increase in caspase 3 activation in treated cells. All these results confirm a significant induction of apoptosis via intrinsic pathway by oxaliplatin [99].

Based on these evidences and on previous studies, Gourdier and collaborators evidenced, through RNA interference assay, that a lack of expression of Bax and/or Bak (another member of Bcl-2 family) proteins does not allow the mitochondrial apoptotic process to start in colorectal carcinoma cells exposed to oxaliplatin. In this way, the authors demonstrated that the presence of Bax and Bak is probably an essential step in oxaliplatin-induced intrinsic apoptosis activation [100].

Since adducts can associate with several proteins, including mitochondrial proteins such as VDAC, a structural compound of mPTP that, if altered, can modify the mitochondrial permeability, Xiao and collaborators tested the olesoxime, an inhibitor of VDAC opening, in combination with oxaliplatin *in vivo*. Through behavioral tests they observed that a prophylactic treatment with olesoxime significantly improves the peripheral nervous system damage. The authors also examined a preventive treatment with ALC, a protective compound for mitochondria, reaching the same results [38,97].

**Figure 3:**
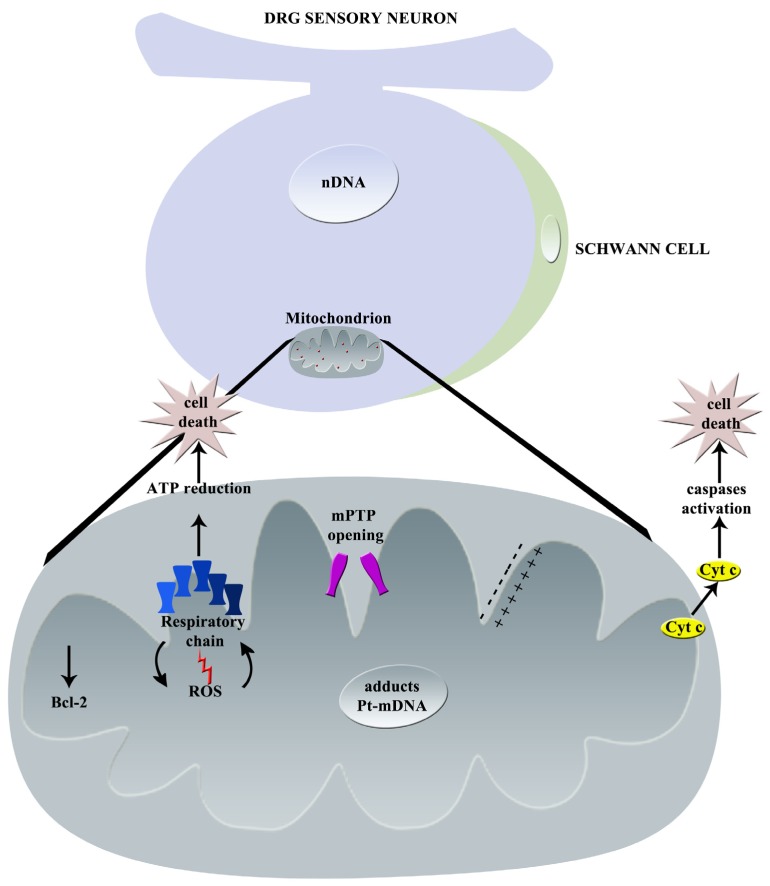
Graphical summary of oxaliplatin-induced mechanisms of neurotoxicity: principal effects on mitochondrion (nDNA = nuclear DNA, Pt = platinum, mDNA = mitochondrial DNA, Cyt c = Cytochrome C, ROS = Reactive Oxygen Species, mPTP = mitochondrial Permeability Transition Pore).

The principal effects of oxaliplatin on mitochondria are graphically summarized in Figure 3.

## 5. Vincristine

Vincristine is an important anticancer drug mainly employed in the treatment of hematologic cancers and pediatric sarcomas. Vincristine, as well as other Vinca alkaloids, is known to impair microtubule dynamics, assembling and disassembling, resulting in cell cycle arrest at metaphase [101]. *In vitro* experiments performed during the 80s/early 90s demonstrated that vincristine stabilizes microtubules by binding their ends and by inhibiting the hydrolysis of GTP (guanosine triphosphate) [102,103,104]. Lobert also demonstrated that vincristine has a higher binding affinity with microtubules compared to other Vinca alkaloids and that it induces the formation of paracrystalline aggregates [101].

### 5.1. Vincristine-Induced Peripheral Neuropathy

Most patients in treatment with vincristine present symptoms of peripheral neurotoxicity, which becomes one of the main reasons for treatment discontinuation. They develop disturbances in both motor and sensory functions [105] with early numbness, tingling in hands and feet and ankle jerks [106]. Moreover, neuropathic and muscle pain is frequently present, as well as the loss of temperature sensation. Vincristine mechanism of action that causes the impairment of β-tubulin assembly leads to severe alterations in axonal microtubules, axonal swelling and myelinated and unmyelinated fiber damage [107,108]. The onset of peripheral neuropathy usually occurs at the dose of 4–10 mg and the severity of symptoms is related to the duration and the therapeutic doses received by patients: severe neuropathy occurs at a cumulative dose of 15–20 mg [105,109]. A third of the patients also develop autonomic disorders as orthostatic hypotension, constipation, paralytic ileus, bladder dysfunction and impotence [106]. Sometimes, after discontinuation, the symptoms worsen instead of improving [110]. At the neurophysiological examination, nerve conduction studies show decreased amplitudes of compound muscle and sensory action potentials but no changes in conduction velocities [111].

### 5.2. Vincristine and Mitochondria

Several studies have demonstrated that alterations in mitochondria can be related to the development of vincristine-induced peripheral neuropathy and neuropathic pain.

In 2006, Siau and colleagues demonstrated that vincristine-caused neuropathic pain, an evident mechanical allodynia and mechanical hyperalgesia in rats, was significantly improved by different Ca^2+^ regulators (TMB-8, Quin-2, EGTA and EGTA-AM) [10]. In fact, it was demonstrated that vincristine is able to affect Ca^2+^ movement through the mitochondrial membrane, reducing both the amount and rate of Ca^2+^ uptake and decreasing Ca^2+^ efflux [112]. Alterations in the mitochondrial Ca^2+^ uptake would affect the spatio-temporal changes of Ca^2+^ concentration, alter Ca^2+^ signaling, Ca^2+^ signals [113], and regulate Ca^2+^-dependent processes as increased exocytosis of neurotransmitters [114]. These changes can lead to impaired neuronal excitability and glial function. These alterations are also supported by morphological observation that reported a great incidence of swollen and vacuolated mitochondria with disrupted cristae localized at the periphery of the organelle [25].

Moreover, Joseph and Levine in 2006 demonstrated that also inhibitors of mETC complexes I, II, III, IV, and V (inhibitors of ATP-dependent mechanisms) are able to significantly attenuate vincristine-related neuropathic pain in rats. These experiments demonstrate that mETC contribution in neuropathic pain is ATP dependent [115].

**Figure 4:**
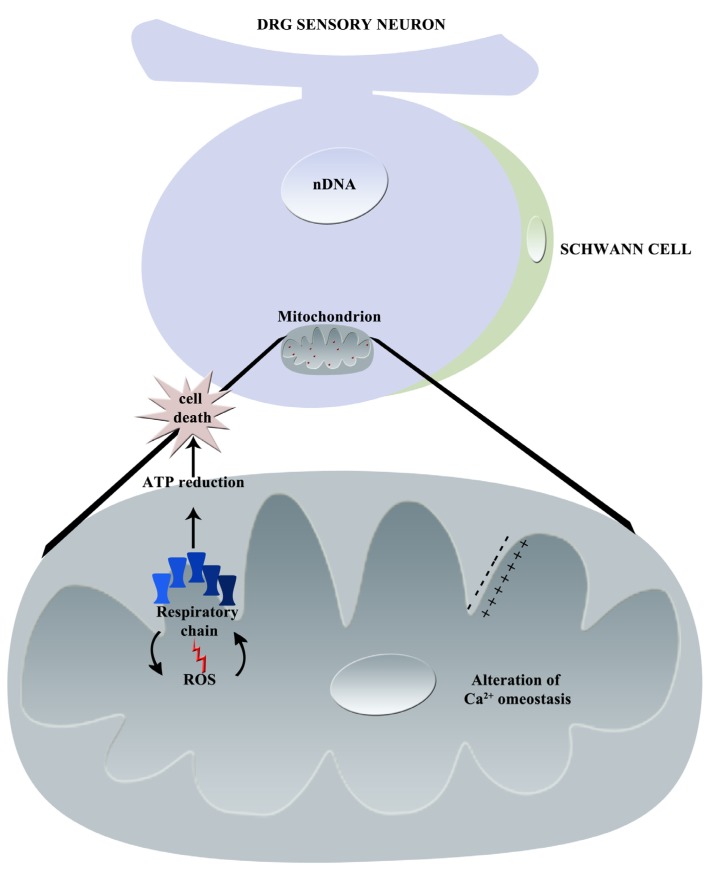
Graphical summary of vincristine-induced mechanisms of neurotoxicity: principal effects on mitochondrion (nDNA = nuclear DNA, ROS = Reactive Oxygen Species, Ca^2+^ = calcium).

In Figure 4, the principal effects of vincristine on mitochondria are graphically summarized.

## 6. Bortezomib

Bortezomib is the first therapeutic proteasome inhibitor employed in humans. It is principally used to treat relapsed multiple myeloma and mantle cell lymphoma [116]. Now, it is tested for other hematological and solid tumors mostly in combination with other drugs [117]. Bortezomib acts by inhibiting the proteasome-ubiquitination pathway [116] by specifically and reversibly binding to the 26S subunit of the proteasome, [118] leading to cell cycle inhibition and apoptosis [119]. One of the proposed explanations for this mechanism of action is the decreased activation of nuclear factor kappa-light-chain-enhancer of activated B cells (NF-κB) that leads to cell death [120].

### 6.1. Bortezomib-Induced Peripheral Neuropathy

Despite its efficacy, its clinical use is often limited by the onset of a painful peripheral neuropathy [121,122] with severe symptoms: paresthesia, burning sensations, dysesthesias, numbness, sensory loss, reduced proprioception and vibratory sensation [123] are some of the most common symptoms referred by patients. Bortezomib can also cause a decrease in the deep tendon reflexes and in the autonomic innervation in the skin [124]. Patients showed axonal degeneration in peripheral nerves [125] while alterations in the DRG are also observed in animal models of bortezomib-induced peripheral neuropathy [9]. The symptoms identified by patients often occurred during the first cycles of treatment but did not increase further after the fifth cycle of therapy [126]. In most patients, peripheral neuropathy is reversible and it is not affected by type and number of previous treatments [126].

### 6.2. Bortezomib and Mitochondria

In 2013, Staff and coworkers demonstrated that bortezomib induces abnormalities in microtubule polymerization in cultured DRG neurons [127]. Moreover, the authors showed that, as a consequence, axonal transport was impaired as happened with antitubulinic chemotherapy [128]. Using a time lapse imaging of mitochondria labeled with TMRM (tetramethylrhodamine methyl ester) in live DRG neurons, they demonstrated that bortezomib induces a decrease in the frequency of mitochondrial movements along neurites. This probably happens because abnormal mitochondrial dynamics negatively influence metabolically-active long neuritic processes, causing a preferential susceptibility to insults and dysfunctions in axonal transport [127]. In chronically bortezomib treated rats, it was demonstrated that the main pathological events observed in DRG and peripheral nerves were represented by mitochondrial damage, more evident in satellite cells than in DRG sensory neurons. This seemed to be associated with an enlargement of the endoplasmic reticulum [9]. These results agree with previous considerations of bortezomib effects in killing myeloma cells by activating the mitochondrial based (“intrinsic”) apoptotic pathway [129,130,131].

Moreover, it was demonstrated that bortezomib induces a dysregulation of Ca^2+^ homeostasis leading to cell apoptotic death. In fact, the endoplasmic reticulum is the main intracellular Ca^2+^ storage and mitochondria also participate in Ca^2+^ homeostasis. Bortezomib, in fact, determines some alterations in the expression of proteins associated with the endoplasmic reticulum secretory pathway, activation of caspase 12, Ca^2+^ homeostasis dysregulation and, finally, cell death [132].

Zheng and collaborators also quantitatively demonstrated that bortezomib-treated rats have defects in mitochondrial function, measured as a significant deficit in ATP production rate following stimulation of the electron transport system [39]. These alterations appeared as early as three days after the last bortezomib dose and lasted, with about the same severity, for one month. Authors hypothesized that the lack of response to the addition of Cyt c in the respiration assays suggests no involvement of mitochondria with damaged membranes as well as the absence of effects on citrate synthase levels and no alterations in the number of mitochondria [39].

Moreover, Zhang demonstrated that a prophylactic treatment with ALC prevents bortezomib-induced deficits in mitochondrial respiration and ATP production, and also neuropathic pain observed in rats [39].

To corroborate these observations, a clinical genetic study performed in 2010 demonstrated that the genetic profiles of patients with early-onset bortezomib-induced peripheral neuropathy present alterations also in genes involved in AMPK-mediated signaling, responsible for the regulation of cellular ATP supply. As an example, CPT1C encodes for an enzyme found in neuron mitochondria that is involved in transport of hydrophobic fatty acid chains into mitochondria, and plays a part in mitochondrial dysfunction [27].

**Figure 5:**
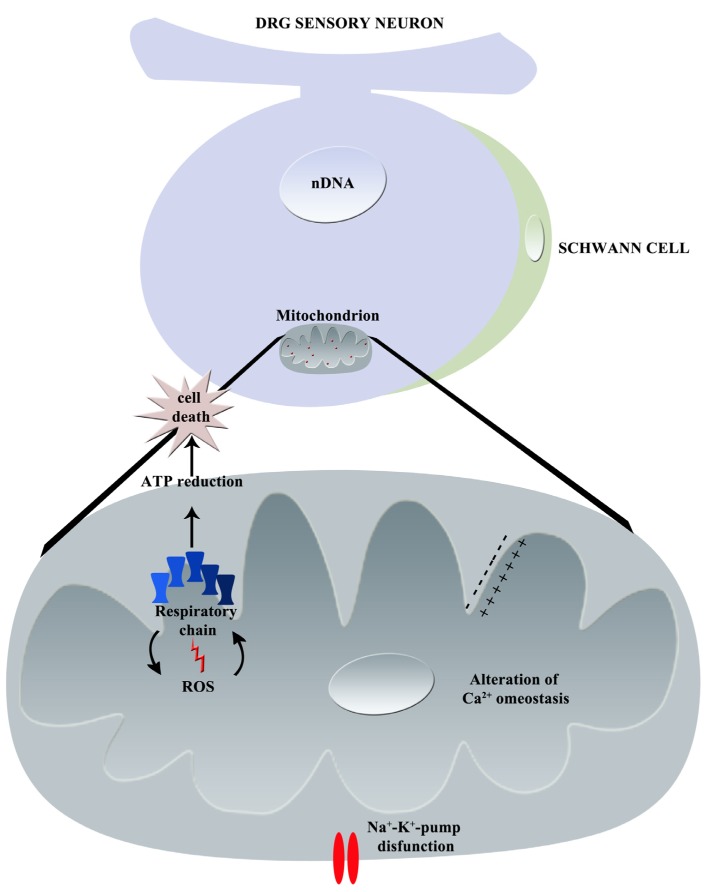
Graphical summary of bortezomib-induced mechanisms of neurotoxicity: principal effects on mitochondrion (nDNA = nuclear DNA, ROS = Reactive Oxygen Species, Ca ^2+^ = calcium, Na^+^ = sodium, K^+^ = potassium).

Nasu in 2014, showed that bortezomib induces sensory-dominant axonal depolarization in humans prior to the development of axonal degeneration. This can lead to nerve hyperexcitability also consistent with positive symptoms such as pain and paresthesia typically observed in bortezomib-treated cancer patients. The axonal membrane depolarization can be explained by several mechanisms including the decrease of the Na^+^/K^+^-ATPase-dependent pump function, or altered Na^+^ or K^+^ conductance [133]. However, the possibility that the depolarizing shift of membrane potential is due to an impairment of the Na^+^/K^+^-ATPase-dependent pump and ensuing Na^+^ axonal accumulation [134,135] is more reliable, considering that one of the main bortezomib actions is on mitochondria. This can be explained by a continuous influx of Na^+^ ions that causes an overload of the Na^+^/K^+^-ATPase-dependent pump, resulting in mitochondrial energy conversion failure [133].

In Figure 5, the principal effects of bortezomib on mitochondria are graphically summarized.

## 7. Conclusions

Antineoplastic drugs with different chemical structures and mechanisms of action often cause the development of peripheral nervous system disorders, which can include axonal and/or myelin damage, DRG structural abnormalities and related functional deficits. Multiple unique and common molecular mechanisms can be responsible for different aspects of their neurotoxicity. Among the common mechanisms, alterations of the structural integrity and/or the functionality of mitochondria can be responsible for determining the onset, development and severity of CIPN.

In Table 2, the principal effects of chemotherapeutic drugs on mitochondria are summarized.

**Table 2:** Summary of some of the principal effects of chemotherapeutic drugs on mitochondria in the peripheral nervous system.

Drugs	Principal actions on mitochondria [references]
**PACLITAXEL**	alteration of mitochondrial Ca^2+^ homeostasis [60,62]
	alteration of mitochondrial permeability (vacuolization and swelling by mPTP pore modification) [25,63,64]
	alteration of mitochondrial respiratory chain (deficit in respiration and in ATP production) [38]
	induction of apoptotic mitochondrial pathway (by Cyt-c release and Bcl-2 inactivation) [66]
**CISPLATIN**	mDNA-Pt adducts formation [79]
	alteration of mitochondrial respiratory chain (deficit in respiration and in ATP production) [84]
	ROS production [71,86,87,88]
	induction of apoptotic mitochondrial pathway (by Cyt c release and caspases activation) [89,90,91,92]
**OXALIPLATIN**	mDNA-Pt adducts formation [41]
	alteration of mitochondrial respiratory chain (deficit in respiration and in ATP production) [38]
	alteration of mitochondrial permeability (by mPTP pore) [38,97]
	induction of apoptotic mitochondrial pathway (by Cyt c release and Bcl-2 activation) [99,100]
**VINCRISTINE**	alteration of mitochondrial Ca^2+^ homeostasis [112,113,114]
	alteration of mitochondrial respiratory chain [115]
**BORTEZOMIB**	alteration of mitochondrial Ca^2+^ homeostasis [132]
	alteration of mitochondrial respiratory chain (deficit in ATP production) [39]
	impairment of Na^+^/K^+^-ATPase-dependent pump [133,134,135]
